# Usefulness of the Clinical Frailty Scale in patients with end-stage kidney disease

**DOI:** 10.1093/ckj/sfae132

**Published:** 2024-05-02

**Authors:** Kyra Lamberink, Yolande M Vermeeren, Arthur D Moes, Jeanette Mulderij, Paul A Rootjes, Tizza P Zomer

**Affiliations:** Department of Internal Medicine, Gelre Hospitals, Apeldoorn, The Netherlands; Department of Internal Medicine, Gelre Hospitals, Apeldoorn, The Netherlands; Department of Internal Medicine, Gelre Hospitals, Apeldoorn, The Netherlands; Department of Internal Medicine, Gelre Hospitals, Apeldoorn, The Netherlands; Department of Internal Medicine, Gelre Hospitals, Apeldoorn, The Netherlands; Department of Internal Medicine, Gelre Hospitals, Apeldoorn, The Netherlands

**Keywords:** Clinical Frailty Scale, dialysis, end-stage kidney disease, frailty, frailty index

## Abstract

**Background:**

The frailty index (FI) is commonly used to estimate frailty in end-stage kidney disease (ESKD) patients. The Clinical Frailty Scale (CFS) is a less time-consuming alternative. We aimed to determine the test performance of the CFS for pre-dialysis and dialysis patients and patients receiving conservative therapy from the Dialysis Centre Apeldoorn.

**Methods:**

In this cross-sectional study, haemodialysis, peritoneal dialysis, pre-dialysis patients and patients receiving conservative therapy from the Dialysis Centre Apeldoorn were included and subjected to frailty assessment. Nephrologists not familiar with the CFS completed the frailty score after medical consultation. The sensitivity, specificity and area under the curve (AUC) of the CFS were determined. The FI was used as the gold standard.

**Results:**

Included were 144 patients, of whom 60 (41.7%) were considered frail according to the FI. The mean age was 67.4 ± 13.5 years and 56 (38.9%) were female. The cut-off point of the CFS for ‘vulnerable’ (CFS ≥4) had a sensitivity of 63.3%, a specificity of 81.0% and an AUC of 0.72. The cut-off point of the CFS for ‘frail’ (CFS ≥5) had a sensitivity of 50.0%, a specificity of 91.7% and an AUC of 0.71.

**Conclusions:**

The CFS is a quick and easy-to-use tool for the determination of frailty in ESKD patients with a high prevalence of frailty. Nevertheless, the sensitivity of the CFS in the present study was considered too low to implement into daily clinical practice. The sensitivity might be increased by training nephrologists in the use of the CFS.

KEY LEARNING POINTS **What was known:**

- The prevalence of frailty among end-stage kidney disease (ESKD) patients is high.
- Conventional frailty screening, i.e. the frailty index, is complex and time consuming.
- There is a need for an easy-to-use, less-time-consuming tool to implement frailty screening into daily clinical practice.

**This study adds:**

- The prevalence of frailty is 41.7% in all ESKD patients and 55.6% in ESKD patients treated with haemodialysis.
- The Clinical Frailty Scale (CFS) with the vulnerable cut-off point (≥4) had a sensitivity of 63.3% and a specificity of 81.0%.
- The CFS with the frail cut-off point (≥5) had a sensitivity of 50.0% and a specificity of 91.7%.

**Potential impact:**

- The CFS is an easy and less-time-consuming tool to assess frailty.
- The sensitivity of the CFS was considered insufficient to be implemented into daily clinical practice when used by untrained nephrologists.
- More research is necessary to improve the sensitivity of the CFS.

## INTRODUCTION

Frailty is a condition that is characterized by the dysregulation of multiple physiological systems, resulting in a reduction in physiologic reserve and increased vulnerability to morbidity and mortality [1, 2]. The prevalence of frailty is high among patients suffering from end-stage kidney disease (ESKD). A study conducted by Drost *et al.* in 2016 [3] revealed that 36.8% of ESKD patients were considered frail, with an even higher prevalence of 43.6% observed among patients >65 years of age. Frailty can result in various adverse patient outcomes, including disability, loneliness, diminished quality of life (QoL), depression, increased hospitalization rate and nursing home admission [2, 4]. Consequently, identification of frailty is important, as it may influence decisions regarding treatment and end-of-life care [5].

Advance care planning (ACP) aims to improve end-of-life care and is already implemented as the standard of care for several diseases associated with frailty [6, 7]. Many studies on ACP have shown promising results, including a reduction of in-hospital deaths, a shorter duration of hospitalization, a reduction in depression and increased satisfaction of both patients and medical care personnel [6, 7]. Moreover, ACP turned out to have a positive impact on the family members of dialysis patients. A pilot study in the United States examined the impact of ACP and end-of-life care in dialysis patients and their surrogates. The implementation of ACP resulted in increased consistency of decision-making between patient and surrogates. Moreover, surrogates had more confidence in making decisions regarding end-of-life care of their relatives. Furthermore, surrogates were less affected by anxiety, depression and post-traumatic stress symptoms after the death of a relative [8]. Despite these promising outcomes for patients and their families, ACP is currently not implemented as the standard of care in ESKD patients in the Netherlands.

Before ACP can be widely implemented in ESKD patients, there is the need for a simple and time-efficient tool to assess which patients can benefit (most) from ACP [9, 10]. As frailty is a dynamic condition, it is essential to regularly assess frailty status. Screening tools such as comprehensive geriatric assessment and the frailty index (FI) have already been applied within the field of nephrology, however, these are all time-consuming and complex to use and therefore not immediately applicable for daily clinical practice [9, 11]. Furthermore, the prevalence of frailty should be assessed to estimate what resources (personnel and time) are needed to be allocated for ACP.

The Clinical Frailty Scale (CFS) is a clinical assessment tool that is easy to use and takes very little time to complete. The CFS consists of nine categories, where patients with a score of 4 are considered vulnerable and a score of 5 are considered frail [12]. The CFS has already been used in multiple fields, including geriatric medicine, cardiology, intensive care and the emergency department [13]. This tool can quickly determine the patient's frailty status to provide appropriate care, such as ACP.

Although the existing literature provides insights into the validation of the different frailty screening tools among the different treatment modalities of ESKD patients [9, 11, 14–16], so far no study has examined implementation of the CFS compared with the FI in haemodialysis (HD) and peritoneal dialysis (PD) patients, patients receiving conservative therapy and in pre-dialysis patients. Thus it is unknown whether the CFS can be used as a daily clinical frailty screening tool for all ESKD patients.

Therefore, the primary aim of this study was to determine the test performance of the CFS for pre-dialysis, dialysis patients and patients receiving conservative therapy from the Dialysis Centre Apeldoorn in order to assess the usefulness of the CFS in daily clinical practice. The secondary aim was to determine the frailty prevalence to estimate what resources are needed for ACP.

## MATERIALS AND METHODS

A cross-sectional observational study was conducted among ESKD patients from October 2021 until October 2023 in the Dialysis Centre Apeldoorn. The study did not fall under the scope of the Dutch Medical Research Involving Human Subjects Act (WMO). A non-WMO declaration was provided by the Medical Ethics Committee of Isala Hospital, Zwolle, Netherlands (number 210614). Written informed consent was obtained from all participants.

### Frailty index

The FI and CFS were used to assess frailty. In this study, the FI was calculated according to the method of Searle *et al.* [17], which examines the ratio of deficits to the total number of components. The FI consists of 38 frailty components that are categorized into the following groups: activities of daily living (ADL), physical aspects, psychosocial factors, comorbidities and functional tests. The functional tests consisted of grip strength, usual walking speed, rapid walking speed and cognitive functioning measured by the Mini Mental State Examination (MMSE). A patient with a FI score ≥0.25 was considered frail. The absence of a deficit was scored as 0 and the presence of a deficit as 1. The values 0.25, 0.5 and 0.75 were used as intermediates. Missing data were excluded from the FI and the FI score was subsequently calculated as the sum of the available components [17].

As part of the FI, comorbidities were determined using the standard operating procedure of the Charlson Comorbidity Index (CCI) [18]. Apart from inquiring about the patient's most recent outdoor walking session, cycling was also considered an acceptable part of the physical component of the FI.

With respect to the 6.1-m walking test, patients walked their normal walking speed and rapid walking speed twice. The fastest time of each test was used to measure frailty [17]. The cut-off time limit for a normal walking pace was <16 s and <10 s for a rapid walking pace [17]. Above these cut-off values, patients scored 1, indicating frailty. For safety reasons, some patients did not execute the rapid walking test. Nevertheless, if their normal walking pace was still <10 s, the rapid walking test was scored as 0, indicating no frailty.

Grip strength was determined by calculating the average of three measurements on the preferred hand using a hydraulic hand dynamometer (Saehan, Changwon, South Korea) [3]. Unrecordable grip strength and inability to walk were scored as 1, as it is a predictor for frailty.

### Clinical Frailty Scale

The CFS, introduced by Rockwood *et al.* [12], is a far easier tool used to determine frailty and has been translated into Dutch [19]. The CFS is visual and contains a brief description of each of the nine classes, ranging from very fit to terminally ill. Patients who scored 4 on the CFS scale were considered vulnerable, as they have symptoms that limit activities, but they are not dependent on others for daily help. A patient with a score ≥5 was considered frail, often because of impairments in ADL. The research nurse asked the treating nephrologist to classify the patients after medical consultation. Unlike the FI, the patient does not require any functional tests since the classification relies solely on the clinical judgement of the nephrologist. The nephrologists had no specific training or experience prior to utilization of the CFS.

### Inclusion and exclusion criteria

Patients were considered eligible if they had a estimated glomerular filtration rate (eGFR) <20 ml/min/1.73 m^2^ calculated by using the Chronic Kidney Disease Epidemiology Collaboration (CKD-EPI) equation, visited the Dialysis Centre Apeldoorn and were ≥18 years of age. Patients were excluded if they were unable to speak Dutch, unable to provide informed consent or if the treating physician or nurse decided the burden of study participation was too high due to limited life expectancy.

### Statistical analysis

Descriptive statistics concerning patient characteristics were collected. For continuous variables, mean ± standard deviation (SD) or median [interquartile range (IQR)] were calculated. Categorial variables were presented as absolute and relative (%) frequencies.

The sensitivity, specificity, positive predictive value (PPV) and negative predictive value (NPV) of the CFS were calculated using 2 × 2 tables. The FI was considered the gold standard to assess frailty. Test accuracy was analysed using a receiver operating characteristics (ROC) curve to measure the area under the curve (AUC). An AUC of 0.5, according to the Hosmer–Lemeshow test, indicated no discrimination between the two tests, an AUC of 0.7–0.8 was considered as acceptable discrimination, an AUC of 0.8–0.9 was considered excellent and an AUC >0.9 was outstanding [20]. The level of significance was set at .05.

Depending on the distribution, grip strength and walking time were analysed by a Student's *t*-test or Mann–Whitney U test. Variables with skewness values between −1 and 1 were assumed to be normally distributed [21]. All statistical analyses were performed using the Statistical Package for Social Sciences version 28 (IBM, Armonk, NY, USA).

## RESULTS

### Study population

**Figure 1:**
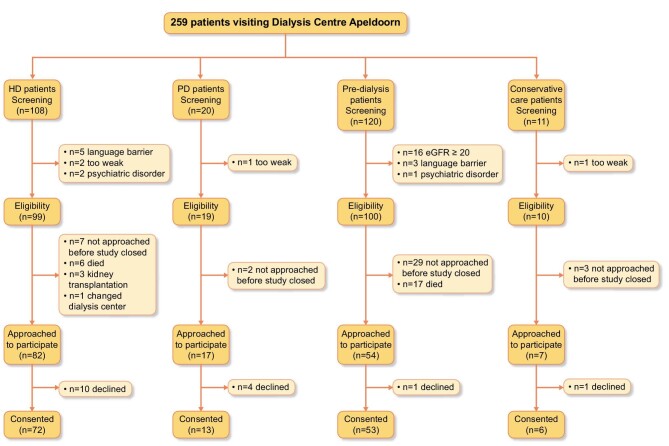
Flow chart of study participation.

The study flow chart is presented in Fig. 1 and the patient characteristics are provided in Table 1. In short, a total of 144 patients (response rate 90.0%) were included. The mean age of all patients was 67.4 ± 13.5 years and 56 (38.9%) patients were female. Overall, 72 patients (50.0%) were treated by HD, 13 (9.0%) by PD, 6 (4.2%) received conservative treatment and 53 (36.8%) were pre-dialysis patients.

**Table 1:** Patient characteristics.

Characteristics	Number (%) N = 144
Female, *n* (%)	56 (38.9)
Age (years), mean ± SD	67.4 ± 13.5
<65, *n* (%)	51 (35.4)
≥65, *n* (%)	93 (64.6)
Caucasian, *n* (%)	130 (90.3)
Treatment modality, *n* (%)	
Pre-dialysis care	53 (36.8)
Conservative therapy	6 (4.2)
PD therapy	13 (9.0)
HD therapy	72 (50.0)
Daytime HD	62 (86.1)
Night-time HD	10 (13.9)
BMI, median (IQR)	26 (23.0–29.7)
eGFR (ml/min/1.73 m^2^), median (IQR)	8.0 (6.0–13.8)
Time on dialysis (months), median (IQR)	27 (5.8–64.8)
No dialysis, *n* (%)	62 (43.1)
<12 months, *n* (%)	32 (22.2)
>12 months, *n* (%)	50 (34.7)
Hospitalizations last year, median (IQR)	0 [0–1]
Medications, mean ± SD	13.2 ± 6.4
Charlson Comorbidity Index, median (IQR)	2 (1–3)

### Frailty

**Figure 2:**
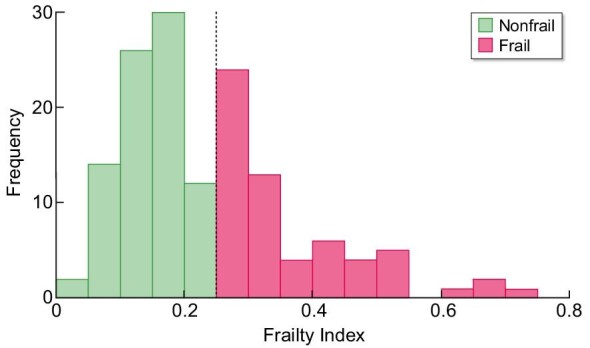
Frequency of frailty measured with FI with reference line at 0.25.

**Figure 3:**
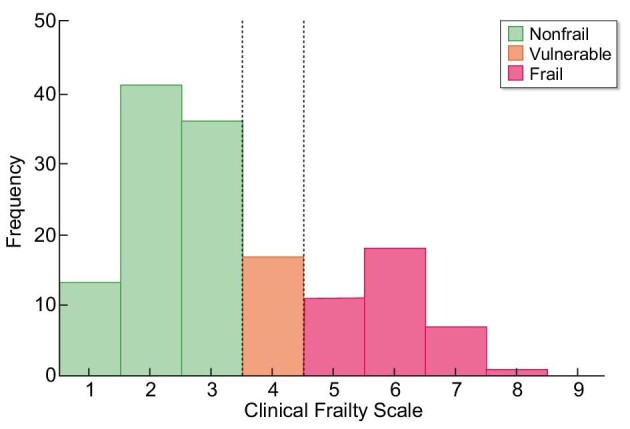
Frequency of frailty measured with the CFS with reference lines (CFS 4 and CFS 5).

The results of the FI and CFS are shown in Figs. 2 and 3. Table 2 presents the FI and CFS scores for different subgroups. According to the FI, a total of 60 (41.7%) patients were considered frail. The frailty prevalence was 50.5% for patients ≥65 years of age and 25.5% for the younger group. The CFS identified 37 (25.7%) patients as frail and 17 (11.8%) patients as vulnerable. Taking into account the different subgroups, 55.6% of the HD patients were frail according to the FI, compared with 20.8% of the pre-dialysis patients.

**Table 2:** Comparison of frailty in patients with different treatment modalities.

Characteristics	All patients (*N* = 144)	Pre-dialysis (*n* = 53)	HD (*n* = 72)	PD (*n* = 13)	Conversative therapy (*n* = 6)
Frail by FI	60 (41.7)	11 (20.8)	40 (55.6)	5 (38.5)	4 (66.7)
Frail by CFS ≥4	54 (37.5)	14 (26.4)	34 (47.2)	3 (23.1)	3 (50.0)
Frail by CFS ≥5	37 (25.7)	7 (13.2)	27 (37.5)	3 (23.1)	0 (0)

Data are presented as *n* (%).

### Screening instruments

Table 3 presents the sensitivity, specificity, AUC, PPV and NPV of the CFS. The cut-off point for vulnerable (CFS ≥4) had a sensitivity of 63.3% and a specificity of 81.0%. The frail cut-off point (CFS ≥5) had a lower sensitivity (50.0%) but higher specificity (91.7%). The PPV was lower for the vulnerable cut-off point (70.4% versus 81.1%) and the NPV was higher for the vulnerable cut-off point (75.6% versus 72.0%). The AUC for the vulnerable cut-off point was 0.72 [95% confidence interval (CI) 0.63–0.81] and for the frail cut-off point it was 0.71 (95% CI 0.62–0.80).

**Table 3:** Comparison of screening instruments for frailty using the FI as the gold standard.

Screening instrument	CFS ≥4	CFS ≥5
Sensitivity, % (95% CI)	63.3 (49.9–75.4)	50.0 (36.8–63.2)
Specificity, % (95% CI)	81.0 (70.9–88.7)	91.7 (83.6–96.6)
AUC (95% CI)	0.72 (0.63–0.81)	0.71 (0.62–0.80)
PPV, % (95% CI)	70.4 (59.5–79.4)	81.1 (66.9–90.1)
NPV, % (95% CI)	75.6 (68.5–80.6)	72.0 (66.4–76.9)

**Figure 4:**
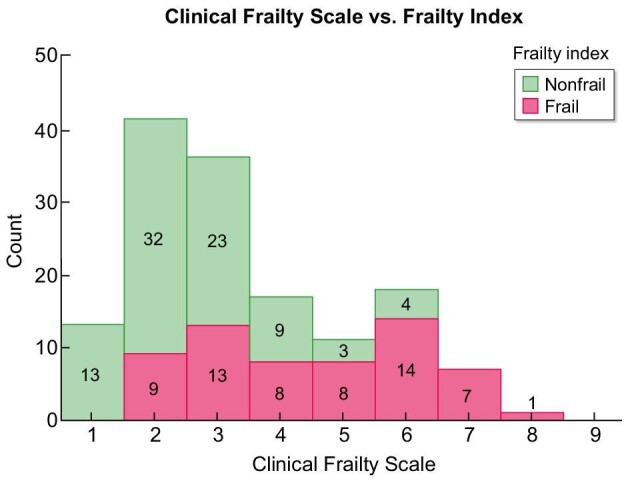
Frailty frequency of the CFS compared with the FI.

Figure 4 presents the frailty categorization of the CFS compared with the FI. Within the second category of the CFS, nine individuals were classified as nonfrail. However, based on the FI, they were considered frail and therefore false negative. The same applies for 13 patients in CFS class 3. In contrast, a total of seven individuals were classified as frail in CFS classes 5 and 6, despite not being considered frail by the FI. These patients were false positive.

### Functional tests

The mean grip strength for frail women was 15.4 kg and 20.0 kg for nonfrail women (*P* = .007). Frail men had an average grip strength of 25.6 kg and 36.3 kg in nonfrail men (*P* < .001). Two patients were unable to perform the grip strength test as a result of arthrosis or muscle spasms. Additionally, two patients did one of three attempts during the grip strength test due to a painful shunt and four patients did two of three attempts due to pain or spasms.

Regarding the walking test, frail patients needed 7.1 s to complete the normal walking test compared with 5.6 s in nonfrail patients (*P* < .001). Furthermore, for the rapid walking test, frail patients needed more time than nonfrail patients (4.5 s versus 3.6 s; *P* < .001). A total of 20 patients were unable to complete the normal and rapid walking tests due to a wheelchair (*n* = 15), instability (*n* = 3) or pain (*n* = 2). Additionally, 14 patients were unable to perform the rapid walking test due to instability (*n* = 13) or shortness of breath (*n* = 1). However, of these 14 patients, 11 completed the normal walking test in <10 s and thus did not score positive for frailty on the rapid walking test. Four patients used a walking stick and eight patients used a wheeled walker.

The FI included eight comorbidities [median score 2 (range 0–6)]. Arthritis was the most frequent comorbidity.

Missing values for calculating the FI were rare. Seven patients (4.9%) missed one component and two patients (1.4%) missed two components of the FI. Consequently, the FI for these patients was determined by dividing the sum of components through 37 or 36 instead of the usual 38 components.

## DISCUSSION

The current study provides valuable insights into the frailty status of ESKD patients and assessing the usefulness of the CFS as a daily clinical screening tool for frailty in ESKD patients. According to the FI, 41.7% of all ESKD patients were frail, which is consistent with previous findings [3, 4, 11]. When divided into younger and older patients with ESKD, our current findings are also in line with other studies [3, 11]. Notably, frailty was also prevalent among pre-dialysis patients.

The most remarkable finding of our current study is the low sensitivity of the CFS (63.3% with a cut-off ≥4 and 50.0% with a cut-off ≥5) to detect frail patients. This means that approximately half of all frail ESKD patients will not be detected when using the CFS. On a positive note, the specificity of the CFS is remarkedly higher (81.0% with a cut-off ≥4 and 91.7% with a cut-off ≥5). According to the Hosmer–Lemeshow test, the CFS is an acceptable discriminative test (AUC = 0.72 with a cut-off ≥4 and AUC = 0.71 with a cut-off ≥5) [20].

A possible explanation for the low sensitivity of the CFS in detecting frailty is the lack of training in treating nephrologists. Moreover, the current consultation procedure is unprepared to determine the CFS score, as no specific questions were formulated up front to inquire about patients ADL condition. In addition, after medical consultation the research nurse asked the nephrologist the CFS score of the patient. Therefore, they may not have had the opportunity to inquire about the patient's fitness and ADL status, which is required for the CFS.

Another remarkable finding is the low hospitalization rate of our ESKD patients. This might be due to the frequent contact between ESKD patients and their dialysis nurse and nephrologist. This frequent contact enables early detection of medical problems, thereby reducing hospitalizations.

The prospective nature is the most important strength of our study. The standardized manner in which data were collected, resulting in minimal missing data, is another strength. Additionally, we included both pre-dialysis patients and ESKD patients receiving all forms of dialysis. This is a more diverse patient population compared with previous studies [9, 14–16]. As a result, our findings provide insights into the entire ESKD patient population which enables us to determine who should receive ACP and what resources are required.

An important limitation of this study is that nephrologists were not trained to use the CFS. Additionally, there might be an underestimation of frailty according to the FI, possibly due to the exclusion of patients for whom the burden of the research was too high (n = 4). The FI was used as gold standard. A comprehensive geriatric assessment might be considered a better gold standard. However, for research purpose this was not feasible. The walking test lacked a turning component, resulting in a simpler walking test. Moreover, some patients did not perform the walking test or grip strength test. All these factors may have influenced the FI [17, 22].

The use of the CFS as a fast and simple clinical frailty screening tool is currently not feasible due to its low sensitivity. Nevertheless, it is important to further explore the implementation of the CFS in daily clinical practice. One of the most important reasons is the high prevalence of frailty among ESKD patients. These patients can experience adverse outcomes as a result of their frailty and will likely benefit from more appropriate care, such as ACP. Another reason to further explore use of the CFS is its practical usefulness. Compared with other frailty tools, the CFS is considerably less time-consuming. According to previous studies, assessment of the FI requires a minimum of 40 min per patient [23], whereas the CFS takes <10 min [24]. Our own experience indicates an even greater time difference, with the FI taking at least 1 h and the CFS <5 min. To enhance its performance, an easily applicable classification tree has recently been developed as an adjunct to the CFS. This tree is intended to increase the reliability of the CFS when employed by inexperienced assessors [25]. Given that the nephrologists did not receive any training, this tree could potentially serve as a solution to increase the sensitivity of CFS.

In conclusion, the CFS in its current form was considered insufficient to be implemented into daily clinical practice for the assessment of frailty in an ESKD patient population where frailty is highly prevalent. Perhaps the sensitivity of the CFS might increase by training nephrologists in its use.

## Data Availability

The data underlying this study are available from the corresponding author upon reasonable request.
